# The Improved Milk Quality and Enhanced Anti-Inflammatory Effect in Acetylserotonin-O-methyltransferase (*ASMT*) Overexpressed Goats: An Association with the Elevated Endogenous Melatonin Production

**DOI:** 10.3390/molecules27020572

**Published:** 2022-01-17

**Authors:** Hao Wu, Xudai Cui, Shengyu Guan, Guangdong Li, Yujun Yao, Haixin Wu, Jinlong Zhang, Xiaosheng Zhang, Tuan Yu, Yunxiang Li, Zhengxing Lian, Lu Zhang, Guoshi Liu

**Affiliations:** 1 National Engineering Laboratory for Animal Breeding, Key Laboratory of Animal Genetics and Breeding of the Ministry of Agricultural, Beijing Key Laboratory for Animal Genetic Improvement, College of Animal Science and Technology, China Agricultural University, Beijing 100193, China; 18800160525@163.com (H.W.); gsy729@cau.edu.cn (S.G.); 15600911225@cau.edu.cn (G.L.); yujunyao1995@163.com (Y.Y.); wuhaixin@cau.edu.cn (H.W.); lianzhx@cau.edu.cn (Z.L.); luzhang2018@cau.edu.cn (L.Z.); 2 Qingdao Senmiao Industrial Co., Ltd., Qingdao 266101, China; xdcui5188@163.com (X.C.); yxli@samuels.cn (Y.L.); 3 Tianjin Institute of Animal Husbandry and Veterinary, Tianjin 300192, China; jlzhang1010@163.com (J.Z.); zhangxs0221@126.com (X.Z.); 4 Tianheng Animal Health and Product Quality Supervision Station, Qingdao 266200, China; fchdongjianzhan@126.com

**Keywords:** acetylserotonin-O-methyltransferase (*ASMT*), melatonin, goat, cytokines, intestinal microbiota, arachidonic acid, milk quality

## Abstract

Background: Transgenic animal production is an important means of livestock breeding and can be used to model pharmaceutical applications. Methods: In this study, to explore the biological activity of endogenously produced melatonin, Acetylserotonin-O-methyltransferase (*ASMT*)-overexpressed melatonin-enriched dairy goats were successfully generated through the use of pBC1-ASMT expression vector construction and prokaryotic embryo microinjection. Results: These transgenic goats have the same normal phenotype as the wild-type goats (WT). However, the melatonin levels in their blood and milk were significantly increased (*p* < 0.05). In addition, the quality of their milk was also improved, showing elevated protein content and a reduced somatic cell number compared to the WT goats. No significant changes were detected in the intestinal microbiota patterns between groups. When the animals were challenged by the intravenous injection of *E. coli*, the *ASMT*-overexpressed goats had a lower level of pro-inflammatory cytokines and higher anti-inflammatory cytokines compared to the WT goats. Metabolic analysis uncovered a unique arachidonic acid metabolism pattern in transgenic goats. Conclusions: The increased melatonin production due to *ASMT* overexpression in the transgenic goats may have contributed to their improved milk quality and enhanced the anti-inflammatory ability compared to the WT goats.

## 1. Introduction

Melatonin affects immune system function and the nervous system through endocrine effects. [1]. Melatonin is mainly secreted by the pineal gland of vertebrates at night [2], but other tissues and cells, including the immune cells [3], skin cells [4], and gastrointestinal tract cells, also synthesize melatonin [5]. Melatonin has a strong antioxidant ability that can slow down aging, suppress tumor growth, and boost immune activity [6]. Thus, the therapeutical effects of melatonin have been tested in many disease-associated models, including neurodegenerative diseases. Melatonin treatment effectively retards the processes of Alzheimer’s disease [7], multiple sclerosis [8], and amyotrophic lateral sclerosis [9]. Additionally, melatonin is regularly used to treat primary sleep disorders and sleep disorders related to neuronal diseases in children [10,11]. In fact, all the disorders mentioned above are more or less associated with imbalanced immunity and inflammation [12]. Inflammation is a complex pathophysiological process, and the dynamic balance of pro-inflammatory and anti-inflammatory mediators in the body maintains inflammatory homeostasis [13]. Inflammation causes the excessive production of reactive oxygen species (ROS) and reactive nitrogen (RNS), initiating oxidative stress [14]. As a potent antioxidant, melatonin can effectively reduce oxidative stress [15]. In animal studies, an elevated melatonin level is mostly associated with improved anti-inflammatory activity [16]. In this respect, melatonin can suppress pro-inflammatory cytokines and enhances anti-inflammatory cytokines under different pathophysiological conditions [17,18].

Melatonin synthesis in organisms involves several enzymes. Among them, the arylalkylamine N-acetyltransferases (*AANAT*) and Acetylserotonin-O-methyltransferase (*ASMT*) are considered to be rate-limiting enzymes [19]. In *AANAT*-overexpressed dairy goats, high levels of endogenous melatonin render their anti-inflammatory ability by regulating autophagy processes [20]. *ASMT* is the last rate-limiting enzyme in melatonin synthesis and also plays an important role in melatonin synthesis. For example, the upregulation of *ASMT* expression in the pineal gland of rats is associated with increased melatonin synthesis [21]. The *ASMT* gene demonstrates significant polymorphism, and its gene promoter and first exon polymorphism may be related to mental disorders [22]. On other hand, the Single Nucleotide Polymorphism (SNP) of *ASMT* is involved in the improvement of reproductive performance in goats [23].

It has been reported that goat milk contains a greater variety of oligosaccharides has higher level of nutritional value than cow milk [24]. Recombinant human antithrombin III (RHAT III) functional goat milk has been successfully developed by using somatic cell nuclear transfer technology [25]. This technology has been widely used to generate transgenic livestock [26]. For example, transgenic sheep with the overexpression of *AANAT* and *ASMT* in the mammary glands have been reported [27]. By using prokaryotic embryo microinjection technology, Deng et al. successfully generated TLR4 overexpression in sheep with the enhanced clearance of invaded microbes [28].

In the current study, we explored the effects of *ASMT* overexpression on the milk quality and fatty acid (arachidonic acid) metabolism in the anti-inflammatory response of transgenic animals compared to WT animals. The results will provide novel information that will allow us to further understand the biological traits modified by melatonin-enriched transgenic technology in animals, particularly in goats.

## 2. Results

### 2.1. Production and Identification of ASMT Overexpressed Dairy Goats

**Figure 1:**
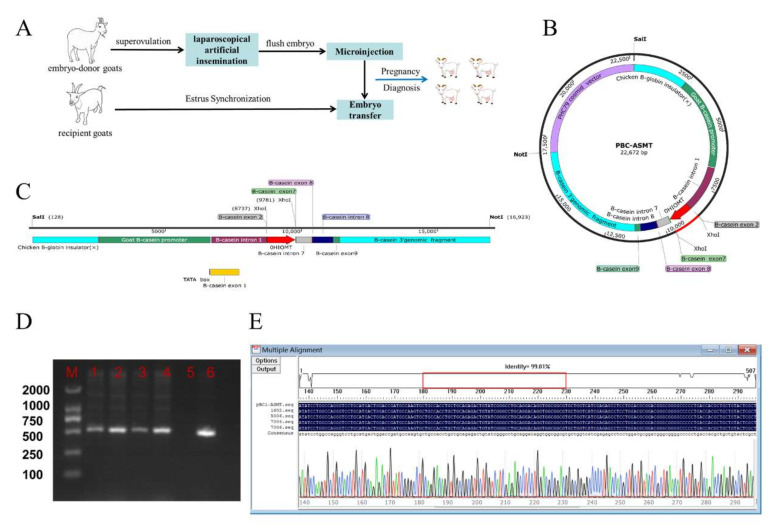
The procedure to generate *ASMT*-overexpressed goats. (**A**)The process used to design prokaryotic embryo microinjection; (**B**) pBC1-ASMT vector; (**C**) micro-injection vector of ASMT; (**D**) PCR identification of transgenic offspring (M: marker, 1:1602, 2:5006, 3:7004, 4:7006, 5: control, 6: pBC1-ASMT); (**E**) sequence alignment of transgenic offspring.

**Figure 2:**
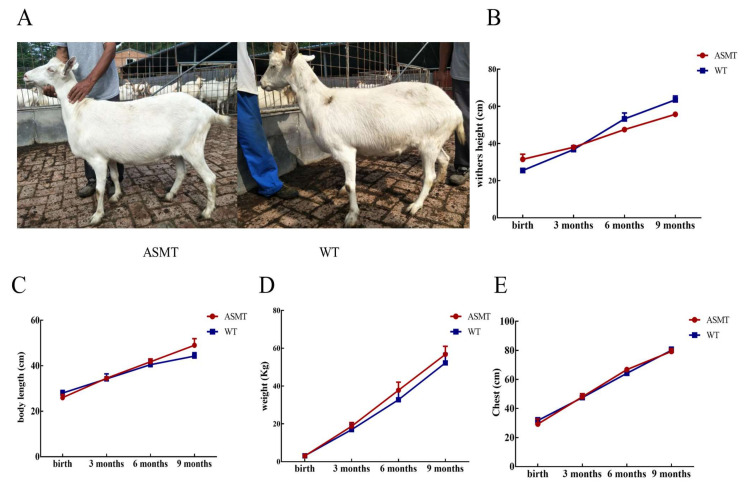
The general phenotype of *ASMT*-overexpressed goats compared to the WT goats. (**A**) Photos of *ASMT*-overexpressed and WT goats; (**B**) the body height; (**C**) the body length; (**D**) The body weight; (**E**) the breast size. Note: the transgenic goat (*n* = 3), wild type (*n* = 3).

The Coding DNA Sequence (CDS) region of the *ASMT* gene was cloned from the pineal gland of a goat for the construction of mammary gland-specific expression vector pBC1-ASMT (Figure 1B). SaII and NotI endonucleases were used to obtain linearized fragments for microinjection (Figure 1C). A total of 241 embryos were obtained by artificial insemination with superovulation from 21 donor Laoshan dairy goats. A total of 222 embryos were obtained after the microinjection of the linearized fragments. A total of 55 recipients were transplanted, and 18 were pregnant (Figure 1A). PCR sequencing was performed on 10 female lambs, and 4 lambs were transgenic positive, with a positive rate of 40% (4/10) (Figure 1D,E). There were no significant differences observed in the growth measurement between the *ASMT*-overexpressed dairy goats and the WT goats (*p* > 0.05) (Figure 2).

### 2.2. Determination of Melatonin Levels in Blood and Milk and Analysis of the Milk Quality

**Figure 3:**
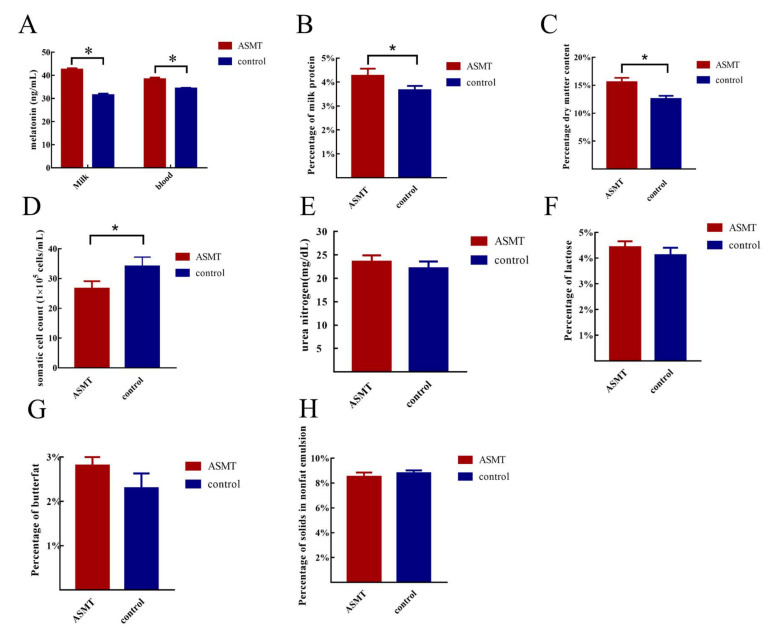
Comparisons of the melatonin levels in blood and milk as well as the milk parameters between transgenic (ASMT) and WT goats. (**A**) Melatonin levels in the blood and milk; (**B**) protein content in milk; (**C**) dry matter content in milk; (**D**) somatic cell count in milk; (**E**) urea nitrogen in milk; (**F**) lactose content in milk; (**G**) fat in milk; (**H**) non-lipid solids in milk. Note: the transgenic goat (*n* = 3), wild type (*n* = 3), * represents significant differences between the two groups.

The melatonin levels in the blood and milk of the *ASMT*-overexpressed goats were significantly higher than that of the WT goats (*p* < 0.05) (Figure 3A). The milk quality of the *ASMT*-overexpressed dairy goats was further analyzed. The protein and dry matter contents in the milk were significantly increased (*p* < 0.05) (Figure 3B,C) and the somatic cell number was significantly decreased in the transgenic goats compared to in the WT goats (*p* < 0.05) (Figure 3D). There were no significant differences in terms of the milk fat, lactose, non-fat milk solids, and urea nitrogen contents between the *ASMT*-overexpressed and WT goats (*p* < 0.05) (Figure 3E–H).

### 2.3. Microbiota Distribution Analysis

**Figure 4:**
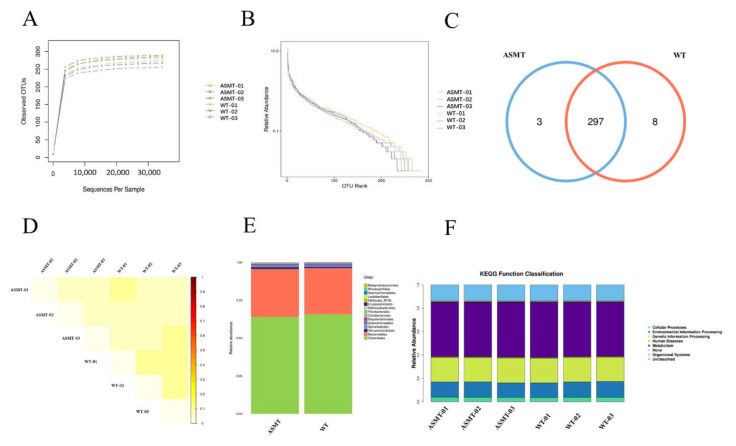
Gut microbiota distributions in transgenic (ASMT) and WT goats. (**A**) Dilution curve; (**B**) rank–abundance curve; (**C**) Venn diagrams; (**D**) distance matrix heat map; (**E**) histogram of distribution of species at the level of order; (**F**) heat map of KEGG Function Classification at the order level. Note: the transgenic goat (*n* = 3), wild type (*n* = 3).

To further evaluate the health status of the *ASMT*-overexpressed goats, 16S sequencing was performed to detect their fecal microbiome distribution. With the increase in the effective sequence depth, the dilution curve of the sample increased rapidly and then leveled off, which indicated good sequencing quality (Figure 4A). With the increase in the rank value, the decreasing trend in the abundance curve was gradually stable, which indicated that the uniformity and richness of the species in the sample were of good quality (Figure 4B). There was no significant difference in the number of Operational Taxonomic Units (OTUs) after sequencing between the *ASMT*-overexpressed dairy goats and the WT goats (*p* > 0.05) (Figure 4C). β-diversity analysis showed a small difference coefficient and a small difference in species diversity between the two groups (Figure 4D). Further analysis of microbial sequencing at the order level showed that the two groups of goats were mainly concentrated in Bacteroidales and Clostridiales (Figure 4E). The Kyoto Encyclopedia of Genes and Genomes (KEGG) pathway was mainly enriched in metabolic and genetic information processing functional areas, and there was no significant difference between the *ASMT*-overexpressed dairy goats and the WT goats (*p* > 0.05) (Figure 4F). Thus, the fecal microbiome distribution of the *ASMT*-overexpressed dairy goats was normal, which was also the case in the WT goats.

### 2.4. Changes in Body Physiological Indexes after E. coli Injection

**Figure 5:**
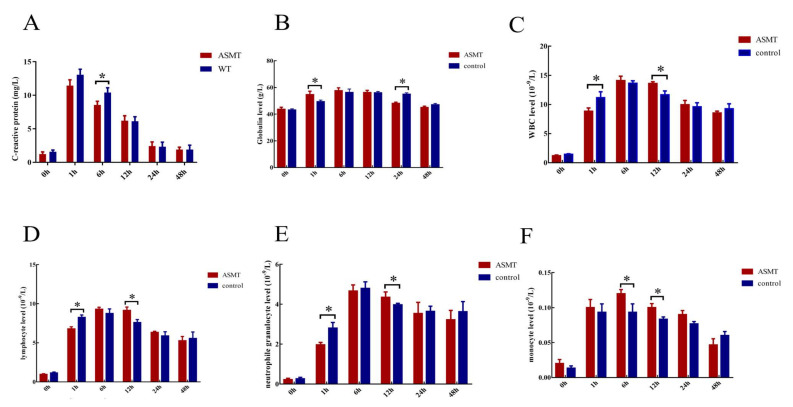
The effects of *E. coli* injection on the biophysiological indexes in transgenic (ASMT) and WT goats. (**A**) C-reactive protein content; (**B**) globulin content; (**C**) white blood cell content; (**D**) lymphocyte content; (**E**) neutrophil content; (**F**) content of monocyte macrophages. * represents significant differences between the two groups.

To explore the *ASMT*-overexpressed dairy goats in terms of their inflammatory response, 500 µL of *E. coli* (10^7^/mL) was injected into the jugular vein. As a marker of inflammatory response, the C-reactive protein (CRP) increased rapidly within 1 h and then gradually decreased. The CRP level in the *ASMT*-overexpressed dairy goats was significantly lower than that in the WT goats (*p* < 0.05) (Figure 5A). No significant differences were observed in the globulin levels between the two groups (*p* > 0.05) (Figure 5B). The leukocyte level at 1 h after *E. coli* injection was significantly higher in the *ASMT*-overexpressed goats than it was in the WT goats, while at 12 h, it was significantly lower than it was in the WT group (*p* < 0.05) (Figure 5C). No significant differences were observed in the lymphocyte or neutrophil levels between the two groups (*p* > 0.05) (Figure 5D,E). The level of mononuclear macrophages was higher in the *ASMT*-overexpressed goats than it was in the WT goats (*p* < 0.05) (Figure 5F).

### 2.5. Changes of Inflammatory Biomarkers

**Figure 6:**
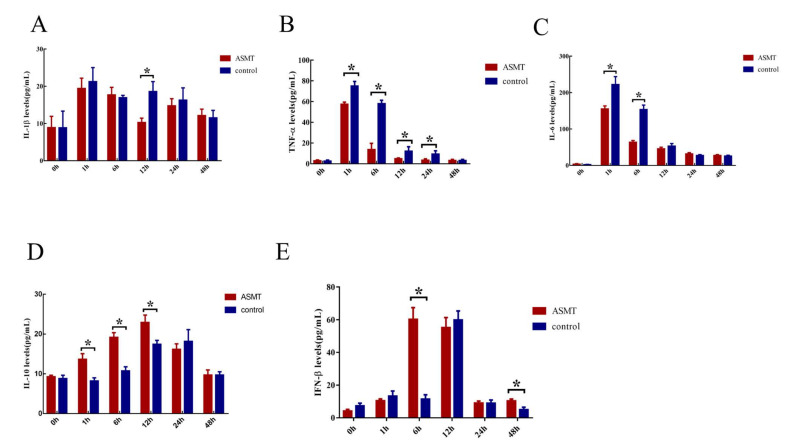
The effects of *E. coli* injection on inflammatory cytokines in transgenic (ASMT) and WT goats. (**A**) IL-1β; (**B**) TNF-α; (**C**) IL-6; (**D**) IL-10; (**E**) IFN-β. Note: the transgenic goat (*n* = 3), wild type (*n* = 3), * represents significant differences between the two groups.

The proinflammatory cytokines IL-1β, TNF-α, and IL-6 rapidly reached a peak after 1 h after *E. coli* injection and then decreased gradually in both groups. The level of the proinflammatory cytokines in the *ASMT*-overexpressed goats was significantly lower than that in the WT goats (*p* < 0.05) (Figure 6A–C). The key anti-inflammatory factors IL-10 and interferon β (IFN-β) were gradually increased after *E. coli* injection and decreased after 12 h. The levels of the anti-inflammatory factors in the *ASMT*-overexpressed goats were significantly higher than they were in the WT goats 1–12 h after *E. coli* injection (*p* < 0.05) (Figure 6D,E).

### 2.6. Serum Metabolism Analysis after E. coli Injection

**Figure 7:**
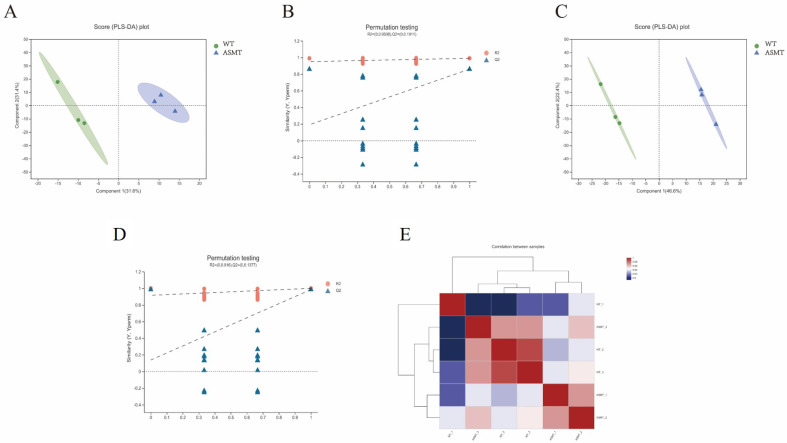
PLS-DA and heat map analyses in transgenic (ASMT) and WT goats. (**A**) Positive ion score (PLS-DA) chart; (**B**) positive ion array detection; (**C**) negative ion score (PLS-DA) chart; (**D**) anion array detection; (**E**) metabolite heat map analysis. Note: the transgenic goat (*n* = 3), wild type (*n* = 3).

After *E. coli* injection, the Partial Least Squares Discriminant Analysis (PLS-DA) method was used to analyze the serum metabolomic alterations. The confidence level of the PLS-DA data was above 95%, and different groups were in different ellipses. For the analysis of the positive ions, two principal component analysis groups were obtained (Figure 7A). The cumulative interpretation rate R2X (cum) and R2Y (cum) of the PLS-DA model for principal component 1 were 31.6% and 95.2%, respectively, and the cross-validation predictive power of the model was 66.1% (q2 cum). The cumulative interpretation rates of principal component 2 (R2X) and R2Y (cum) were 31.4% and 99.1%, respectively, and the cross-validation predictive power of the model was 58.6% (Q2 cum). With the decrease in replacement retention, R2 and Q2 decreased, and the regression line showed an upward trend, which indicated that the prediction model was reliable (Figure 7B). The analytic results of the anions were similar to cations (Figure 7C,D). The cationic and anion data showed significant differences, indicating that there was a significant difference in the serum metabolome between the *ASMT*-overexpressed dairy goats and the WT goats (*p* < 0.05). Heat map analysis also showed significant differences in the metabolic composition and abundance between the groups (*p* < 0.05) (Figure 7E).

### 2.7. ASMT Overexpression Associated Metabolic Consequences

**Figure 8:**
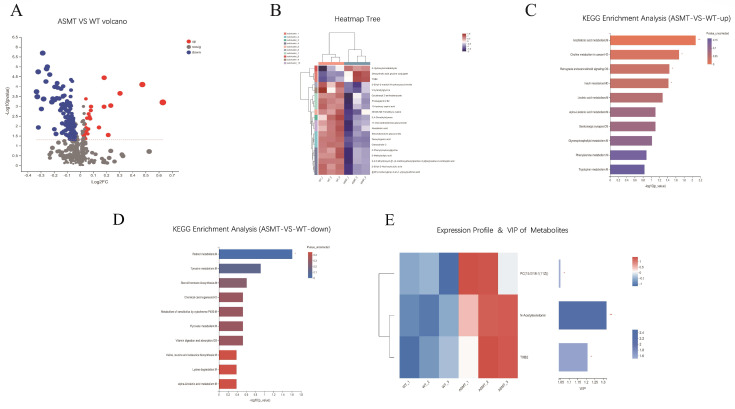
PLS-DA Metabolic analysis of transgenic (ASMT) and WT goats. (**A**) Volcanic map of differential metabolites; (**B**) clustering heat map of differential metabolites; (**C**) KEGG functional analysis of cationic; (**D**) KEGG function analysis of anion; (**E**) VIP analysis diagram of differential metabolites. Note: the transgenic goat (*n* = 3), wild type (*n* = 3). * represents *p* < 0.05, ** represents *p* < 0.01.

In the transgenic animals, the serotonergic synapse and tryptophan metabolism pathways are up-regulated, as identified by the KEGG enrichment analysis (Figure 8C). The retinol metabolism pathway, tyrosine metabolism, and steroid hormone biosynthesis were significantly down-regulated (*p* < 0.05) (Figure 8D) in the *ASMT*-overexpressed dairy goats compared to the WT goats. Based on the KEGG of the up-regulated genes, VIP analysis was performed on the substances in the arachidonic acid metabolism, serotonergic synapse, and tryptophan metabolism pathways. The N-acetylserotonin VIP value was 1.32, and the TXB2 value was 1.21 (Figure 8E). N-acetylserotonin has the largest VIP, which can act as a metabolic marker. N-acetylserotonin is the key substrate of *ASMT*. A total of 274 cations were detected, of which 28 were up-regulated, and 276 anions were detected, of which 138 were down-regulated (Figure 8A). The differential metabolites in the serum metabolome of the goats were analyzed by heat map clustering (Figure 8B). Deoxycholic acid glycine conjugate and TXB2 were significantly up-regulated in the *ASMT*-overexpressed goats compared to in the WT goats (*p* < 0.05). Furthermore, the up-regulated and down-regulated genes were enriched for KEGG function. After the enrichment of the up-regulated gene pathways, arachidonic acid metabolism, choline metabolism in cancer, retrograde endocannabinoid signaling, and insulin resistance showed significant differences between the groups (Figure 8C).

## 3. Discussion

Using transgenic technology to produce livestock with targeted traits has become an important research area in molecular breeding [29]. Prokaryotic embryo injection and somatic cell nuclear transfer are the key methods for the production of transgenic livestock [30]. These methods also have great medical utility. For example, the transgenic animal mammary gland bioreactors can produce high quality recombinant immuno-proteins for the treatment of human diseases at a low cost [31]. The antithrombin III factor has been successfully isolated from transgenic goat milk for clinical use [32]. Melatonin is a potent antioxidant that can retard aging, regulate biological rhythm, and increase anti-inflammatory and immune activities [33]. *AANAT* transgenic goat models have been generated by using somatic cell nuclear transfer. *AANAT*-overexpressed goats with high endogenous melatonin levels showed good anti-inflammatory ability when challenged by LPS [25]. *ASMT* is the last rate-limiting enzyme in the melatonin synthetic pathway [34]. In this study, *ASMT*-overexpressed dairy goats were successfully produced. In addition, several biological traits of these transgenic goats were investigated, including the quality of their milk. In fact, these dairy products make up an important part of the human diet and are a part of a healthy life. Goat milk is more similar to human milk in terms of its nutritional factors than cow milk is [35]. The number of somatic cells in milk is the decisive factor to determine the quality of the milk [36]. The high numbers of somatic cells in milk indicate mastitis, and this milk is not suitable for drinking [36]. However, melatonin supplementation in Holstein cows reduces the number of somatic cells in milk and improves milk quality [37]. Melatonin also lowers milk fat synthesis in bovine mammary epithelial cells by inhibiting mTOR pathway activation [38]. pBC1-ASMT, which was used in this study, is a mammary gland-specific expression vector; thus, *ASMT* will only be expressed in the mammary gland. As predicted, the level of melatonin in milk is significantly increased in transgenic goats compared to in WT goats. This indicates that the construction of the model of *ASMT* mammary gland-specific-expression transgenic goats was successful. To evaluate the quality of the milk, milk composition analysis, which is a commonly accepted index that is used to evaluate the milk quality [39], was performed. The analysis showed a significant decrease in the number of somatic cells in the milk of transgenic goats compared to the WT goats. The data are consistent with previous observations in Holstein cows [37]. In addition, the proteins and dry matter content in the transgenic goat milk were also significantly increased compared to in the milk from the WT goats. All of these results indicate the improved milk quality produced by the transgenic goats compared to the WT goats. The results suggest the feasibility of using *ASMT* overexpression to produce high-quality goat milk.

In the current study, we observed that mammary-specific *ASMT*-overexpression goats has significantly increased levels of circulating melatonin. This is not surprising since lipophilic melatonin molecules can diffuse freely from the mammary gland into circulation. As a result, it can also increase the melatonin level in the gut. Due to the fact that melatonin involves the gut–microbiome–immune axis and regulates the biorhythm through intestinal bacterial activity signals mediated by the NF-Kβ pathway [40], whether *ASMT* overexpression would influence the gut microbiota was also investigated. It was reported that melatonin supplementation reprograms the structure of the intestinal microflora and improves the circadian rhythm of the intestinal microflora in mice fed with a high-fat diet [41]. However, no studies have investigated the effects of high levels of endogenous melatonin on intestinal flora. In this study, 16S sequencing is used to address this issue. The results showed that there was no significant difference in the bacterial flora structure between the two groups. The dominant bacterial flora were Bacteroidales and Clostridiales in both groups. The KEGG functional enrichment analysis showed that the intestinal microflora in transgenic and WT goats was mainly distributed in functional areas important for metabolic and genetic information processing. This indicates that *ASMT* overexpression has little effect on the normal microbial community in the gut.

Another that was focused on was the anti-inflammatory activity of melatonin-enriched transgenic goats since melatonin is also a potent anti-inflammatory molecule. For this purpose, *E. coli* was injected into the goats to induce inflammation. It has been reported that this injection induces serious inflammatory reactions and triggers brain and liver cell death in chickens [42] and increases the production of the proinflammatory cytokines TNF-α, IL-1β, and IL-6 in mice [43]. In the current study, *E. coli* injection induced an immediate increase in CRP, an inflammation marker in animals. However, this increase in CRP was significantly suppressed in the *ASMT*-overexpressed goats compared to in the WT goats. The most impressive observations were that the serum proinflammatory cytokines IL-1β, TNF-α, and IL-6 were significantly reduced while the anti-inflammatory cytokines IL-10 and IFN-β were significantly increased in the *ASMT* transgenic goats compared to in the WT goats after *E. coli* challenge. These cytokine alterations may be the primary contributors to the anti-inflammatory activity of transgenic goats. On other hand, the altered fatty acid metabolism in the *ASMT*-overexpressed goats may also contribute to anti-inflammatory improvement. PLS-DA analysis indicates that the arachidonic acid (AA) metabolism pathway is the most significant pathway for the enrichment of upregulation factors in *ASMT*-overexpressed goats. AA and its derivatives are involved in immune metabolic and inflammatory reactions in organisms [44]. It has been reported that the derivatives of long-chain polyunsaturated fatty acids such as arachidonic acid and docosahexaenoic acid are important mediators for the regulation of inflammation [45]. Prostaglandin, leukotriene, thromboxane, and other arachidonic acid derivatives are associated with the intensity and duration of inflammation in the body [46]. PGE2, PGD2, and TXB2 are inflammatory mediators. Their suppression can reduce inflammatory reactions [47]. Melatonin significantly reduces the conversion of [3H]-AA to prostaglandin (PG) F2 and thrombatin (Tx) B2 and slightly inhibits the conversion of [3H]-AA to PGE2 and PGD2 [48]. TXB2 is produced by arachidonic acid metabolism in human neutrophils [49]. Physiologically, inflammation is a normal reaction that the body has against injury or pathogen invasion. A proper inflammatory reaction is beneficial to the body when recovering from injury or pathogen infection. However, when inflammation processes are overactive, then they usually cause unnecessary tissue damage or even cell death.

In the current study, we observed that there are no large phenotype differences between *ASMT* transgenic and WT goats, and the transgenic phenotype also does not influence the normal distribution of the gut microbiota. Our results show that the *ASMT* overexpression with increased endogenous melatonin will involve in the overreaction of inflammation induced by intravenously injected *E. coli.* This observation is consistent with previous reports that melatonin only suppresses the overaction of inflammation and can be used in serious infectious diseases [50]. Other metabolic pathways, including the retinol metabolism pathway, tyrosine metabolism pathway, and steroid hormone biosynthesis pathway, are down-regulated in transgenic goats, and these pathways do not participate in inflammation, and the biological consequences of changes in these metabolic pathways are unknown.

In the study, we generated *ASMT*-overexpressed dairy goats for the first time. These goats can produce melatonin-enriched, high-protein, and low somatic cell milk compared to WT goats. The anti-inflammatory effect of the transgenic goats is also significantly improved. This is indicated by the reduced levels of proinflammatory and increased levels of anti-inflammatory cytokines after *E. coli* injection in transgenic goats compared to in WT goats. The underlying mechanism is related to their elevated melatonin production, which is induced by the overexpression of *ASMT* since melatonin is a potent anti-inflammatory molecule. The limitation of the study is the relatively small numbers of the transgenic goats due to the common practical reasons for large animal studies. Thus, this study can be considered as a concept-proof study. Based on the data from this study, we will design further studies with more samples in the near future. Another area of interest that we have noticed is whether the novel biological traits found in these transgenic goats can pass to their offspring. This is another goal for our future studies. Furthermore, using transgenic animals to improve milk quality may be an alternative way to replace antibiotic treatment. As noted earlier, this is a new area to be explored, and more and well-designed studies will be required to address unsolved problems in the future.

## 4. Materials and Methods

### 4.1. Construction of ASMT Overexpressed Vector

Total RNA was extracted from the pineal gland of goat using Trizol reagent (Invitrogen, Carlsbad, CA, USA, 15,596,018), and cDNA synthesis was performed using a cDNA synthesis kit (Takara, Dalian, China, RR047). The complete open reading frame (ORFs) and some upper and downstream non-coding regions of the *ASMT* gene were amplified by PhantaTM super-Fidelity DNA polymerase (Vazyme, Nanjing, China, P501). The PCR product was cloned into the pMDTM19-T vector (Takara, 6013) and was sequenced according to the manufacturer’s instructions. Primers were designed using Premier 5.0, and the same restriction XhoI site was added to the *ASMT* (mRNA KC290950.1) Capra hircus sequence from the National Center for Biotechnology Information (NCBI). A pBC1 Milk Expression Vector Kit (Invitrogen, K270-01) was used as the carrier skeleton. *ASMT* was derived from the T-vector by the XhoI restriction enzyme and was cloned into pBC1 to construct the vector pBC1-ASMT.

### 4.2. Prokaryotic Embryo Microinjection Procedure 

The production process for the *ASMT*-overexpressed goats is shown in Figure 1A. Healthy Laoshan dairy goat ewes with a body weight about 65 kg were selected and primed with progesterone using a controlled internal drug release (CIDR) device (EAZI-BREED^®^ CIDR^®^ Sheep and Goat Device, Auckland, New Zealand) for estrus synchronization. Excellent robust rams were used for artificial semen collection. Superovulation and endoscopic-assisted insemination were carried out on embryo-donor goats. The plasmids were digested with NotI and SalI enzymes, and the linearized DNA was extracted from the gel and was purified with a DNA purification kit (Tiangen, Beijing, China, DP214). The linearized DNA solution was then injected into the cytoplasm of the prokaryotic embryos at a concentration of 10 µg/mL and a volume of 5 pL. A total of 3 to 5 embryos that were in good condition were transferred to one recipient within one hour. A total of 55 recipients were transplanted. After 60 days, a B-ultrasound was performed to examine the pregnancy status of the recipient. A total of 18 recipients became pregnant, and 10 lambed.

### 4.3. Identification of Progeny

DNA was extracted from the ear tissue of the progeny to identify its transgenic status. Specific primers were designed using Premier 5.0 (Table 1). The linearized target fragment of pBC1-ASMT was identified by PCR, and the PCR product was sequenced at the Beijing Sangon Biotechnology Company (Beijing, China) and was compared with the target fragment of pBC1-ASMT.

### 4.4. Melatonin Assay

Blood samples were collected from *ASMT*-overexpressed dairy goats who were 12 months of age. An amount of 5 mL of goat jugular vein blood was collected, placed at 37 °C for 30 min, centrifuged at 3000 r/min for 10 min, and the serum was stored at −20 °C. Milk sample collection followed the same procedure as “Milk quality analysis”. Blood and milk samples were mixed with methanol in a 1:4 portion and were then oscillated in a vortex. After centrifugation (12,000 r/min for 10 min), the supernatant was collected and filtered into with a microporous membrane for use. Then, melatonin detection was carried out in the central laboratory of the Beijing Institute of Animal Science, Chinese Academy of Agricultural Sciences (Beijing, China) using a high-performance liquid mass spectrometer (Agilent1290-G6470, Santa Clara, CA, USA).

### 4.5. Milk Quality Analysis

Milk was collected for 3 consecutive days after 7 days of lactation. Before collection, the milk residues in the nipple were removed and cleaned. Milk with normal color, smell, and viscosity was collected at 16:00 every day. An amount of 40 mL fresh milk was mixed with 2–3 drops of saturated potassium dichromate solution and was stored at 2–7 °C. Milk composition determination was conducted based on the National Dairy Standards Accreditation Laboratory of Beijing Animal Husbandry and Veterinary Station (Beijing, China). The somatic cell count (SCC) was determined by Fossomatic TMFC (Serial No.91755377, Part No.60002326, made in Denmark), which was based on flowcytometry. Milk protein, fat, and dry matter were measured by MilkoScan FT+ (Serial No.91755049, Part No.60027086, made in Denmark), which was based on Fourier transforinfrared spectrum analysis.

### 4.6. 16S rDNA Sequencing and Analysis

Fecal samples were collected from *ASMT*-overexpressed dairy goats that were 12 months of age. Rectal feces were collected in a sterile environment and were frozen with liquid nitrogen. The samples were sequenced by 16S rDNA in Jinweizhi Biotechnology Co., Ltd (Suzhou, China). For the microbiome DNA extraction method, the PowerSoil DNA Isolation Kit (MoBio Laboratories, Carlsbad, CA, USA) (Omega DNA Kit) was used. Primers 338F (5′-ACTCCTACGGGAGgCAGCAGcag-3′) and 806R (5′-GGACTACNNGGG TATctaat-3′) were used to amplify the V3–V4 region of the bacterial 16S rRNA gene. The clean reads of all samples were clustered and classified into the same OTUs with an identity of 97% similarity using the software (Qiime1.9.1, Colorado City, CO, USA). The RDPC lassifier Bayesian algorithm was used for taxonomic analysis, and the community composition of each sample was counted at a certain level. The reference database was the 16S-Silva_132 16S rRNA database (http://www.arb-silva.de/, accessed on 1 November 2019). The rarefaction curve of the Alpha Diversity and PCoA Unifrac distance matrix of the Beta Diversity were calculated using Qiime (version 1.9.1). PICRUSt was used to predict the functional capacity of the microbial community.

### 4.7. Colony-Forming Unit (CFU) Counts

The *E. coli* K12 strain DH5α were cultured in Luria–Bertani (LB) broth at 37 °C. The bacterial growth phase was measured using the absorbance of the bacterial suspension at 600 nm. An optical density (OD) of ~ 0.4 ensured that the bacteria were in the logarithmic growth phase. The number of bacteria was counted by plate counting through serial 10-fold dilutions of the inoculum to the LB agar. The bacteria were suspended and diluted to a concentration of 1 × 10^7^ cells/mL in 0.9% normal saline for use.

### 4.8. Animal Study Design

The healthy goats were divided into WT and transgenic groups (three 4-year-old female dairy goats in each group). An amount of 500 µL of *E. coli* (10^7^/mL) was injected into the jugular vein of the goats. The blood was collected after *E. coli* injection from the jugular vein for physiological, biochemical, immune factor, and metabolomics analysis. The serum was isolated for the different analyses.

### 4.9. Analysis of Physiological and Biochemical Indexes

The physiological and biochemical parameters of the blood taken from the transgenic goats (*n* = 3) and from the wild type goats (*n* = 3) were measured. The blood was collected at 0 h, 1 h, 6 h, 12 h, 24 h, and 48 h after *E. coli* injection from the jugular vein. An amount of 5 mL of goat jugular vein blood was collected. An amount of 3 mL of blood was placed at 37 °C for 30 min and centrifuged at 3000 r/min for 10 min, and the serum was used directly to detect the biochemical parameters. The globulin (Glob) content was measured using an Automatic dry biochemical analyzer (FDCNX500iVC, Fuji Corporation of Japan, Fuji, Japan). And amount of 2 mL of whole blood samples was used for hematological measurements using an automatic hematology analyzer (Sysmex K-1000D, Sysmex Inc., Kobe, Japan). The numbers of neutrophils (NEU), lymphocytes (LYM), monocytes (MONO), and mononuclear macrophages were recorded. CRP was measured using a hypersensitive C-reactive protein assay kit(B2072). The sample detecting procedure was carried out in accordance with the instructions. The intra-batch coefficient of variation (CV) for the kit was 10%, and the inter-batch coefficient of variation (CV) was 15%.

### 4.10. Radioimmunoassay of Inflammatory Cytokines

The inflammatory cytokines from the serum were measured using the radioimmunoassay kits with the double-antibody sandwich ELISA method (BNIBT, Beijing, China) following the manufacturer’s instructions. This method has been successfully used previously to detect the cytokines of mammals including mice [51] and goats [25]. The value was read at 450 nm using a microplate analyzer. Using OD value as the ordinate and standard concentration as the abscissa, a standard curve was generated by regression fitting with computer software. Regression analysis determined the best fitting curve. According to the OD value of the sample, the concentration can be found on the standard curve. Before the formal experiment, the preliminary experiment was conducted using untreated goat serum, and the best absorbance value fitted to the standard calibration curve that was obtained. Standard curve calibration was performed for each factor ELISA test. The values of the samples were parallel to the standard curve for the ELISA analysis. The intra-batch coefficient of variation (CV) for all kits was 10%, and the inter-batch coefficient of variation (CV) was 15%. The kits for each cytokine are listed as follows: IL1β (interleukin,1β), eBioscience, 88-7261; IL6 (interleukin,6), eBioscience, 88-7066; IL10 (interleukin,10), eBioscience, 88-8086; IFN-β (Interferon β), eBioscience, 88-7126; TNF-α (tumor necrosis factor-α); eBioscience, 88-7346.

### 4.11. Metabolome Analysis

The serum sample collection at 1 h was the same as “Analysis of physiological and biochemical indexes”. Serum (100 uL) was mixed with 0.4 mL of a pre-cooled methanol solution containing 20 μg/mL 2-chloro-L-phenylalanine as the internal standard. The mixed sample was ultrasonicated for 30 min (5 °C, 40 kHz), was placed at −20 °C for 30 min, and was then centrifuged for 15 min (13,000 g, 4 °C). The supernatant was collected and dried with nitrogen. The sample was resuspended in 100 µL complex solution (acetonitrile: water = 1:1). The mixture was centrifuged at 13,000 rpm for 10 min at 4 °C, and 100 μL of supernatant was transferred to the auto-sampler vials for HPLC-MS/MS analysis. A quality control (QC) sample was prepared by mixing aliquots from all supernatant samples (10 μL from each sample). The method was validated by analyzing the pooled QC sample. Chromatographic separation of the metabolites was performed on a Thermo UHPLC system equipped with an ACQUITY UPLC HSS T3 (100 mm × 2.1 mm i.d., 1.8 µm; Waters, Milford, CT, USA). The sample injection volume was 2 µL, and the flow rate was set to 0.4 mL/min. The column temperature was maintained at 40 °C. The mass spectrometric data were collected using a Thermo UHPLC-Q Exactive HF-X Mass Spectrometer equipped with an electrospray ionization (ESI) source operating in either positive or negative ion mode. After the UPLC-MS analyses, the raw data were imported into the Progenesis QI 2.3 (Nonlinear Dynamics, Waters, Milford, Massachusetts, MA, USA) for data analysis. The preprocessing results were fed into Simca-P (Ver 14.0, Umetrics AB, Umea, Sweden) for the multivariate statistical analysis.

### 4.12. Data Analysis

The Data were expressed in the form of mean ± standard error. One-way and Two-way variance (ANOVA) were performed followed by Duncan’s test using SPSS software, version 25.0 (IBM SPSS Statistics, Armonk, NY, USA). The melatonin and milk quality data were analyzed by One-way ANOVA. The physiological and biochemical indicator and inflammatory factor data were analyzed by Two-way ANOVA. Additionally, time was the independent variable, and physiological and biochemical indicators and the inflammatory factors were the dependent variables. *p* < 0.05 was considered statistically significant.

## 5. Conclusions

In conclusion, we generated *ASMT*-overexpressed dairy goats. These goats can produce melatonin-enriched, high-protein, and low somatic cell milk compared to WT goats. Additionally, the anti-inflammatory effect of the transgenic goats also improved significantly. Endogenous high expression of melatonin may improve the anti-inflammatory ability of transgenic dairy goats by affecting arachidonic acid metabolism.

## Figures and Tables

**Table 1:** The sequence and length of primers for *ASMT* gene PCR amplification.

Primers Sequence	Tm/°C	Length/bp
F 5′-ATGTCGGGACATCGTCTTTG-3′	58	507
R 5′-CATCAGAAGTTAAACAGCACAGTTAG-3′	58	

## Data Availability

The 16S rRNA microbial sequencing data have been successfully submitted to the National Center for Biotechnology Information. Accession to cite these SRA data: PRJNA746758.

## Sample Availability

Samples of the compounds are not available from the authors.
